# Editorial: Promising and sustainable microbial feedstocks for biotechnological processes

**DOI:** 10.3389/fmicb.2022.973723

**Published:** 2022-07-15

**Authors:** Fernando Pérez-García, Luciana F. Brito, Marta Irla, João M. P. Jorge, Elvira Sgobba

**Affiliations:** ^1^Department of Biotechnology and Food Science, Norwegian University of Science and Technology, Trondheim, Norway; ^2^Department of Biological and Chemical Engineering, Aarhus University, Aarhus, Denmark; ^3^Instituto de Tecnologia Química e Biológica, Universidade Nova de Lisboa Oeiras, Lisbon, Portugal; ^4^Faculty of Science and Technology, Umeå Plant Science Centre, Umeå University Umeå, Umeå, Sweden

**Keywords:** microbial feedstocks, bioprocesses, circular bioeconomy, cell factories, natural resources, new added-value chains, metabolic engineering, synthetic biology

At present, the ever-increasing human population creates economic, social, and environmental demands which are steadily pushing toward the bio-based production of added-value chemicals from alternative, green and sustainable substrates. However, the vast majority of bio-production processes still rely on raw materials which compete with food and feed production industry for resources (Wendisch et al., 2016). In order to avoid the overexploitation of the commonly used raw materials, the development of more efficient and eco-friendly bioprocesses is in the spotlight (Wendisch et al., 2016; Becker et al., 2018). This Research Topic focused on latest research and critical reviews regarding novel, innovative, and green microbial feedstocks, as well as their application in biotechnological processes. Important efforts have been done during the last decades to enable microbial access to alternative substrates from industrial processes side streams (e.g., glycerol), plant-derived matter (e.g., lignocellulosic carbohydrates), C1-substrates (e.g., methanol and CO_2_), and marine resources (e.g., seaweed-derived carbohydrates) (Meiswinkel et al., 2013; Pérez-García et al., 2022; Wendisch et al., 2022). In this Research Topic, Grewal et al. comprehensively reviewed the use of various agro-industrial residues as microbial feedstocks for pigment production. Some examples of those residues are the lignocellulosic components of sugarcane bagasse, rice straw, wheat straw, wheat bran, rice husk, and corncob. Moreover, the depolymerization of carbohydrate polymers in the lignocellulosic hydrolysates releases sugars that can be used in fermentative bioprocesses, such as the pentoses, xylose and arabinose (Wendisch et al., 2016). Several organisms have the ability to directly use the sugars from those hydrolysates, e.g., *Chromobacterium violaceum*. The fermentation of sugarcane bagasse by *C. violaceum* resulted in the production of 0.15 g/L of the pigment violacein (Venil et al., 2017). On the other hand, other organisms need to be equipped with the genetic machinery to enable access to those sugars. A *Corynebacterium glutamicum* strain was engineered for the production of the marine carotenoid astaxanthin from xylose and arabinose as alternative carbon sources (Henke et al., 2018). Additionally, as pointed out by Wendisch et al. within this Research Topic, the nitrogen present in several side streams may be valuable for the production of nitrogen containing compounds such as amino acids and amines (Wendisch et al.). Protein-rich by-products from agri- and aqua-cultures side streams contain, for instance, partially hydrolyzed proteins that can be recovered from treated inedible slaughterhouse wastes, shrimp shell waste, micro and macro algal biomasses, or residual brans, seeds, and grains from different agro-processes (Wendisch et al.). *Escherichia coli* and *C. glutamicum* are commonly used to produce amino acids and related compounds and, while *E. coli* has a wide range of substrates available, in *C. glutamicum* the use of alternative substrates has been extensively engineered. This broadened a substrate scope of *C. glutamicum* while expanding the nitrogen containing product portfolio, including lignocellulosic and algal hydrolysates, amino sugars from the polymer chitin and fats (Wendisch et al.). Furthermore, gaseous and liquid C1 compounds often occur as waste streams and are available at low cost. Hence, the C1 compounds methanol, formic acid, methane and CO_2_ are gaining great interest as inexpensive and renewable microbial feedstocks. Acetogenic bacteria like *Clostridium aceticum* or *Butyribacterium methylotrophicum* can use C1 compounds anaerobically, converting them into acetate and ethanol which could be used further for the synthesis of added-value chemicals (Stark et al.). However, these microorganisms possess biotechnological limitations due to their metabolic constraints and the lack of an efficient genetic toolbox. As reviewed by Stark et al.. in this Research Topic, an approach to cope with this biotechnological challenge would be combining two sequential fermentation steps, either as separate processes or as integrated two-stage bioprocesses. In this case, acetate or ethanol are produced from C1 substrates in anaerobiosis by acetogenic bacteria and subsequently the acetate or the ethanol are used as carbon source by aerobic bacteria yielding new added-value chains. For instance, 3-hydroxypropionic acid was produced by *E. coli* consuming acetate, which was produced from CO_2_ + H_2_ (syngas) by *Moorella thermoacetica* (Lai et al., 2021). In fact, microbial feedstocks containing several fermentable substrates could be better utilized when applying co-cultivation approaches for repurposing complex mixture toward valuable products. Synthetic intra- and interspecific microbial consortia have been applied successfully to improve multi-substrate utilization during fermentations (Sgobba et al., 2018; Pérez-García et al., 2021). As described in the review published in this Research Topic by Sanitá Lima and Coutinho de Lucas, co-cultivation of several strains can reduce production costs, increase the resistance to the contamination, and increase the ability to produce more powerful synergistic enzymatic cocktails. In particular, the application of fungi co-cultivations for lignocellulosic biomass conversion is a powerful tool due to their ability to produce several lignocellulolytic enzymes. Yet, this field is still quite unexploited regarding biotechnological applications (Sanitá Lima and Coutinho de Lucas). On the other hand, microbial isoprenoid production from alternative carbon sources using model and non-model organisms has been greatly explored as Carruthers and Lee reviewed in this Research Topic. Isoprenoids production has been demonstrated by methylotrophic archaea *Methanosarcina acetivorans* and *Methanosarcina barkeri*, the methylotrophic bacterium *Methylorubrum extorquens*, and the methylotrophic yeast *Pichia pastoris* (Carruthers and Lee). Furthermore, the yeasts *Yarrowia lipolytica* and *Rhodosporidium toruloides* as well as the bacteria *Pseudomonas putida* and *Bacillus subtilis*, have been used for the conversion of lignocellulosic biomass into isoprenoids (Carruthers and Lee). Additionally, phototrophic isoprenoid production was shown by direct modification of the native isoprenoid pathway genes of the cyanobacterium *Synechococcus elongatus* (Carruthers and Lee). In this regard, phototrophic organisms have emerged as powerful green cell factories for the sustainable production from CO_2_. For example, Abdallah et al. showed in their work in this Research Topic the application of combinatorial chloroplast and nuclear genome engineering in the microalga *Chlamydomonas reinhardtii* to enhance the production of the sesquiterpenoid patchoulol. The final strain *C. reinhardtii* UVM4 showed an enhanced production of the sesquiterpenoid patchoulol. Here, CO_2_ was delivered to autotrophic cultures by using high concentration of bicarbonate buffer (Abdallah et al.).

Further research in this area is vital to achieve eco-friendly bio-based production. Nevertheless, we hope that the reader will find in this Research Topic a state-of-the-art reference rooted in the sustainability of microbial bioprocesses.

## Author contributions

FP-G wrote the first draft. LFB, MI, JMPJ, and ES provided critical comments and feedback for revisions. All authors agreed on the submitted version.

## Funding

FP-G was funded by the Department of Biotechnology and Food Science at the Norwegian University of Science and Technology. LFB was funded by the Research Council of Norway within ERA CoBioTech, project number 327216. MI was funded by the Department of Biological and Chemical Engineering at the Aarhus University. JMPJ was funded by the Instituto de Tecnologia Química e Biológica at the Nova University Lisbon. ES was funded by the Faculty of Science and Technology at the Umeå University.

## Conflict of interest

The authors declare that the research was conducted in the absence of any commercial or financial relationships that could be construed as a potential conflict of interest.

## Publisher's note

All claims expressed in this article are solely those of the authors and do not necessarily represent those of their affiliated organizations, or those of the publisher, the editors and the reviewers. Any product that may be evaluated in this article, or claim that may be made by its manufacturer, is not guaranteed or endorsed by the publisher.
